# Correlating PSf Support Physicochemical Properties with the Formation of Piperazine-Based Polyamide and Evaluating the Resultant Nanofiltration Membrane Performance

**DOI:** 10.3390/polym9100505

**Published:** 2017-10-13

**Authors:** Micah Belle Marie Yap Ang, Victor Jr. Lau, Yan-Li Ji, Shu-Hsien Huang, Quan-Fu An, Alvin R. Caparanga, Hui-An Tsai, Wei-Song Hung, Chien-Chieh Hu, Kueir-Rarn Lee, Juin-Yih Lai

**Affiliations:** 1 R&D Center for Membrane Technology, Department of Chemical Engineering, Chung Yuan University, Taoyuan 32023, Taiwan; mbmyang@gmail.com (M.B.M.Y.A.); huian@cycu.edu.tw (H.-A.T.); wshung@cycu.edu.tw (W.-S.H.); cchu@cycu.edu.tw (C.-C.H.); jylai@cycu.edu.tw (J.-Y.L.); 2 School of Chemical Engineering and Chemistry, Mapúa University, Manila 1002, Philippines; victorlau@yahoo.com (V.J.L.); arcaparanga@mapua.edu.ph (A.R.C.); 3 Center for Membrane and Water Science & Technology, Ocean College, Zhejiang University of Technology, Hangzhou 310014, China; 4 Department of Chemical and Materials Engineering, National Ilan University, Yilan 26047, Taiwan; 5 Beijing Key Laboratory for Green Catalysis and Separation, College of Environmental and Energy Engineering, Beijing University of Technology, Beijing 100124, China; anqf@bjut.edu.cn; 6 Department of Chemical Engineering, National Taiwan University of Science and Technology, Taipei 10607, Taiwan

**Keywords:** polysulfone, polyethylene glycol, thin-film composite polyamide membrane, interfacial polymerization, nanofiltration

## Abstract

Membrane support properties influence the performance of thin-film composite nanofiltration membranes. We fabricated several polysulfone (PSf) supports. The physicochemical properties of PSf were altered by adding polyethylene glycol (PEG) of varying molecular weights (200–35,000 g/mol). This alteration facilitated the formation of a thin polyamide layer on the PSf surface during the interfacial polymerization reaction involving an aqueous solution of piperazine containing 4-aminobenzoic acid and an organic solution of trimesoyl chloride. Attenuated total reflectance-Fourier transform infrared validated the presence of PEG in the membrane support. Scanning electron microscopy and atomic force microscopy illustrated that the thin-film polyamide layer morphology transformed from a rough to a smooth surface. A cross-flow filtration test indicated that a thin-film composite polyamide membrane comprising a PSf support (TFC-PEG20k) with a low surface porosity, small pore size, and suitable hydrophilicity delivered the highest water flux and separation efficiency (J = 81.1 ± 6.4 L·m^−2^·h^−1^, R_Na2SO4_ = 91.1% ± 1.8%, and R_NaCl_ = 35.7% ± 3.1% at 0.60 MPa). This membrane had a molecular weight cutoff of 292 g/mol and also a high rejection for negatively charged dyes. Therefore, a PSf support exhibiting suitable physicochemical properties endowed a thin-film composite polyamide membrane with high performance.

## 1. Introduction

Polyamide membranes are widely used in solid or fluid separation technologies. Numerous studies have demonstrated the competitiveness of polyamide membranes in nanofiltration (NF) [1,2], forward and reverse osmosis [2,3,4], dialysis [5], pervaporation [6,7,8,9,10], and gas separation [11,12,13]. NF is the most common membrane technology for water or liquid separation, and it is usually applied to industries such as water [14,15,16,17,18,19,20], food [21,22,23,24], textile [25,26,27], mining [28,29,30,31], agricultural [32,33,34], pharmaceutical, and biotechnological industries [35,36,37]. The major advantage of NF is the simultaneous high permeability and sufficient retention for a specific compound. The following characteristics are specific to NF: a pore diameter less than 2 nm; a higher rejection of multivalent ions compared with monovalent ions; a molecular weight (*M*_W_) cutoff range of 150–2000 g/mol for neutral compounds; and neutral and positive ion rejections, depending on the ion size and shape [38].

Interfacial polymerization (IP) is the primary method for fabricating high-performance NF polyamide membranes [39]. A polycondensation reaction of monomers on a porous polymeric support occurs at the interface of two immiscible solutions (aqueous and organic phases). The following factors affect the morphology of the resulting thin film: the surface structure and polarity of the membrane support, the chemistry and concentration of monomer, solvent polarity, catalysts and additives, temperature and time during reaction and curing, and posttreatments [40,41,42,43,44]. Most studies focus on modifying the active layer or optimizing the monomer property and reaction process, rather than determining the effect of membrane support characteristics.

The choice of a membrane support is crucial. For IP, the usual porous substrate is polysulfone (PSf), polyethersulfone (PES), polyacrylonitrile (PAN), polyetherimide (PEI), or polyvinylidene fluoride. Nonsolvent-induced phase separation is the commonly used method for fabricating membrane supports. Factors affecting the membrane characteristics are polymer concentration, additives, and nonsolvent [45,46,47]. Characteristics that affected the fabrication of reverse osmosis membranes through IP (m-phenylenediamine + trimesoyl chloride (TMC)) included pore size, hydrophilicity, and the surface roughness of membrane supports [48,49]. Singh et al. [49] fabricated reverse osmosis membranes in a large scale. They used a PSf membrane support with different pore sizes of 0.07 and 0.15 μm. They found that membranes containing small pore sizes prevented the penetration of polyamide, resulting in thick layers and high salt rejections. Ghosh and Hoek [48] modified the pore structure and hydrophilicity of a PSf support by adding a solvent to the precipitation bath and additives (polyethylene glycol (PEG)-8000, polyvinylpyrrolidone (PVP)-8000, and PVP-40,000 as pore-forming hydrophilic modifiers) to the casting solution. At the end of the IP process, the hydrophobic support containing numerous large pores translated to a highly permeable polyamide layer.

In NF, piperazine (PIP) is the primary monomer. The following studies reported on membranes based on PIP and TMC monomers reacted on different membrane supports. Oh et al. [50] hydrolyzed PAN membranes to form a carboxyl functional group and a hydrophilic surface. The PIP was connected to the carboxyl group of the hydrolyzed PAN by ionic bonding. The composite membrane consisting of hydrolyzed PAN delivered a higher flux than the unmodified PAN. This implies that enhancing the hydrophilicity of PAN and the ionic bonding of PIP with carboxyl groups leads to a high performance of composite polyamide membranes. Misdan et al. [51] varied the physical properties of a PSf substrate by changing the PSf concentration in the dope solution. They obtained an optimum concentration of 15 wt % PSf and 1 wt % PVP in an NMP solvent. Below the optimum concentration, the pore size was large. The membrane support pores block polyamide, leading to low water permeability. At a high dope solution concentration, the membrane prepared from 18 to 20 wt % PSf had a smaller pore size, but the formed polyamide layer was thicker; thus, less water could permeate. The same group of investigators examined various polymers—PSf, PES, and PEI. They prepared a membrane support at the same conditions. The obtained membrane supports differed in hydrophilicity and surface porosity. The composite membrane prepared using PSf was highly cross-linked and extremely compact, resulting in a high salt rejection and a low flux [52].

Previous studies reported on factors that affected the properties of PSf membrane supports. However, there is still a lack of systematic and in-depth research on how the substrate characteristics influence the interfacial polymerization process, the composite membrane properties, and the nanofiltration performance. Herein, we aimed to take the following steps: (1) alter the properties of a continuously fabricated PSf support by adding PEG of different molecular weights (200–35,000); (2) determine the effects of these properties on the formation of polyamide during the IP process; and (3) evaluate the performance of the resulting NF membranes. Adding PEG to the PSf solution is the most convenient method of altering the membrane physicochemical properties. No study has considered varying the *M*_W_ of PEG added to PSf supports. The quantity and size of additives might affect the performance of thin-film composite (TFC) membranes. TMC and PIP (with 4-aminobenzoic acid (ABA)) were the monomers used in this study; PIP:ABA ratio was optimized in our previous study [53].

## 2. Experimental Section

### 2.1. Materials

PSf (UDEL P-3500) was purchased from Amoco Performance Products (Ridgefield, CT, USA); it was washed using deionized water to remove impurities. Nonwoven polyester was provided by Ahlstrom (Helsinki, Finland). PEG200, PEG1k, and PEG35k were purchased from Sigma-Aldrich (Saint Louis, MO, USA). *N*-methyl-2-pyrrolidone (NMP) and *n*-hexane were obtained from Tedia High Purity (Fairfield, OH, USA). PEG20k, PIP, ABA, raffinose pentahydrate, methylene blue, and rose bengal were supplied by Alfa Aesar (Haverhill, MA, USA). PEG10k was acquired from Merck (Darmstadt, Germany). Brilliant blue R, amido black 10B, α-cyclodextrin, and TMC were products of Tokyo Chemical Industry Co. Ltd. (Tokyo, Japan). Na_2_SO_4_ and NaCl were received from Nihon Shiyaku Industries Ltd. (Tokyo, Japan). Deionized water (18.2 MΩ·cm^−1^) produced using a Lotun Technic machine (Lotun Technic Co., Ltd., New Taipei, Taiwan) was used throughout the experiments. Glucose and sucrose were produced by Showa Chemical Co. Ltd. (Taipei, Taiwan).

### 2.2. Fabrication of Thin-Film Composite Membranes

A continuous casting machine (velocity = 2.5 m/s) fed with nonwoven polyester was used to prepare a PSf support through a wet-phase inversion method (coagulation water bath was used). The water coagulation bath temperature was maintained at 30 °C. Table 1 lists the composition and viscosity of the casting solutions. A casting solution containing 16 wt % PSf/NMP was prepared at 60 °C. PEG (=50% of the PSf amount) of varying *M*_W_ was added to the casting solution. The solution was first degassed for at least 12 h before it was poured into the continuous casting machine (casting knife gap = 200 µm). The solution instantly precipitated (within few seconds) upon its contact with water. The PSf supports were washed in a machine overnight and stored in 1 wt % NaHSO_3_ solution prior to the conduct of IP.

Before IP was conducted, a PSf support was washed thoroughly with deionized water. The PSf support was clamped onto an iron plate and impregnated with an aqueous solution containing PIP and ABA (1:1 *w*/*w* %). After 2 min, the excess aqueous solution was removed. When 0.2 wt % TMC/*n*-hexane was contacted onto the PSf surface for 1 min, a thin layer was formed through IP. For further polymerization, the composite membrane was subjected to convective air heat treatment at 50 °C for 10 min [53].

Membrane supports were represented as PSf-PEGX, whereas TFC membranes were designated as TFC-PEGX, where X was the *M*_W_ of PEG. The NF membrane prepared using only PSf (i.e., no PEG was added) was denoted by TFC-0.

### 2.3. Membrane Characterization

Attenuated total reflectance-Fourier transform infrared (ATR-FTIR) spectroscopy (Perkin Elmer Spectrum 100 FTIR Spectrometer, Waltham, MA, USA) verified the formation of a thin polyamide layer on PSf and the entanglement of PEGs in PSf chains. Field emission scanning electron microscopy (FESEM, S-4800, Hitachi Co., Tokyo, Japan) with an acceleration voltage of 3 kV was used to examine the change in the membrane surface and cross-sectional morphologies. Platinum was sputtered on the surface and cross section of the membrane to achieve the minimum conductivity for a valid FESEM observation. The surface roughness was measured using atomic force microscopy (AFM, Bruker, Billerica, MA, USA). The surface roughness was reported as a root-mean-square roughness (Rq). Automatic interfacial tensiometer (PD-VP Model, Kyowa Interface Science Co. Ltd., Niiza-City, Saitama, Japan) was used to determine the water contact angle at room temperature by dropping approximately 5 µL of deionized water onto the membrane surface by using a syringe. The TFC membrane zeta potential was analyzed using a dynamic light scattering instrument (Zeta Nano ZS, Malvern, UK). Samples were cut into 7 mm × 4 mm (L × W) dimensions were attached to a sample holder by using a double-sided tape. The sample holder was connected to a surface zeta potential cell. Then, the cell was inserted into a cuvette until the sample holder, barrel and electrodes are covered by fluid. The fluid (neutral pH) used in this study comprised 200 nm polystyrene particles [54].

The surface porosity, surface pore size, and membrane thickness were estimated using ImageJ software (National Institutes of Health, Rockville, MD, USA). The bulk porosity was determined using a gravimetric method, according to the following equation:

(1) $$Porosity\;\left(\text{\%}\right)=\frac{\omega_{1}-\omega_{2}}{A\times l\times \rho }\times 100\text{\%}$$

where *ω*_1_ and *ω*_2_ are the weight of the wet and dry membrane, respectively; *A* is the membrane effective area; *l* is the membrane thickness; and ρ is the density of the fluid (*n*-propanol = 803 kg/m^3^).

The surface porosity, surface pore size and membrane thickness of the composite membrane were measured. The measurement of bulk porosity was based on a free-standing membrane support (PEG + PSf). Both the composite and free-standing membranes were cast at similar conditions (the frees-standing membrane support was hand-cast on a glass plate).

### 2.4. Membrane Filtration Test

The pure water permeability of PSf membranes was measured using a cross-flow filtration setup, operating at 0.10 MPa and a controlled flow rate of 0.60–0.70 L·min^−1^. A similar setup was used to determine the NF performance of TFC membranes, except that the membranes were pre-pressurized at 0.65 MPa by using deionized water for 1 h. The pure water flux and salt rejection were measured at 0.60 MPa for 5 min. The effective area of the tested membrane was 0.0024 m^2^. The mass of water was collected for 5 min. The pure water *flux* (J) was calculated using the following equation:

(2) $$Flux\;\left(J\right)=\frac{m}{\rho \times A\times t}$$

where *m* (kg) is the mass of permeate collected in 5 min, *A* (m^2^) is the effective area, and ρ is the water density (1 kg/L).

The salt rejection for TFC membranes was evaluated using 1000 ppm of aqueous Na_2_SO_4_ solution. The rejection was computed using the following equation:

(3) $$R\left(\text{\%}\right)=\left(1-\frac{C_{p}}{C_{f}}\right)\times 100\text{\%}$$

where *C*_f_ and *C*_p_ are the salt concentrations of the permeate and feed solution, respectively. These concentrations were measured using a conductometer (Metrohm, Herisau, Switzerland). A standard curve (conductivity vs. concentration) was used as basis for determining the correct concentration of permeate whose conductivity had been measured.

The rejection of neutral organic solutes (glucose, sucrose, raffinose, and α-cyclodextrin) or charged dyes (rose bengal, brilliant blue R, amido black 10B, and methylene blue) was determined using 1000 ppm aqueous sugar solution or 50 ppm aqueous dye solution, respectively, at similar operating conditions. The rejection of neutral solutes as a function of their molecular weights was plotted to determine the molecular weight cutoff (rejection = 90%) of the best membrane. The concentrations of neutral solutes were obtained using a total organic carbon analyzer (vario TOC select, Elementar, Langenselbold, Germany), whereas the concentrations of dyes were acquired using a UV/VIS Spectrometer (Lambda 650 S, Perkin Elmer, Santa Clara, CA, USA). The wavelength of rose Bengal, brilliant blue R, amido black 10B and methylene blue were 550, 550, 618, and 668 nm, respectively. These wavelengths indicated the highest intensity in the spectra.

## 3. Results and Discussion

### 3.1. Chemical Structure of Polysulfone Supports and Thin-Film Composite Membranes

**Figure 1:**
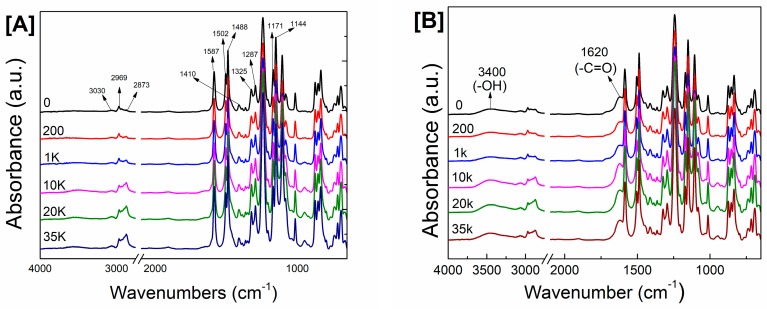
ATR-FTIR spectra of: (**A**) PSf membranes; and (**B**) TFC membranes with varying PEG molecular weights.

Several studies have indicated that adding PEG to a PSf-doped solution improved the surface properties of the resulting membrane [55,56,57,58,59]. Figure 1A presents the ATR-FTIR spectra of PSf supports modified with PEG. Asymmetric and symmetric SO_2_ stretching vibrations were located at 1327–1293 and 1178–1147 cm^−1^, respectively. The stretching of CH_3_–C–CH_3_ was at 1488 cm^−1^. The absorption peaks of C–H aromatic stretching were observed at 3030–3100 cm^−1^, whereas those of C=C aromatic stretching were observed at 1587, 1504 and 1410 cm^−1^. C–H aliphatic stretching in PSf and PEG was at 2969 and 2873 cm^−1^ [60], respectively.

Increasing the PEG *M*_W_ from 200 to 35,000 g/mol increased the PEG absorption peak at 2873 cm^−1^ from 0.0055 to 0.0298 absorbance unit. This apparent peak is due to the entanglement of PEG, which was associated with the increase in viscosity (Table 1) as the additive *M*_W_ increased. This is consistent with previous studies [58]. However, PEGs with a low *M*_W_ readily migrated from the membrane toward the nonsolvent water during the phase-inversion process because of their high solubility in water.

The formation of polyamide layer on different PSf supports was confirmed through ATR-FTIR spectroscopy (Figure 1B). New absorption peaks appeared at 1620 and 3400 cm^−1^, corresponding to amide I (–C=O) and hydroxyl groups, respectively [40,43,60]. As shown in Figure 1B, varying the PEG *M*_W_ apparently had no effect on the change in polyamide absorption peaks.

### 3.2. Effect of the Polyethylene Glycol’s Molecular Weight on Nanofiltration Performance

#### 3.2.1. Properties of Polysulfone Supports Modified Using Polyethylene Glycol of Different Molecular Weights

**Figure 2:**
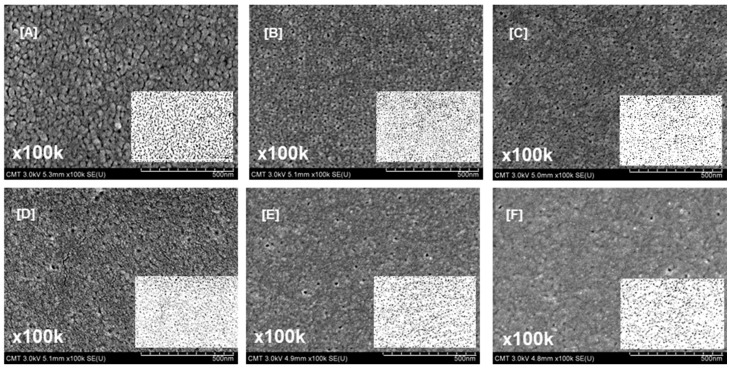
Surface images of PSf supports modified using PEG of varying molecular weights: (**A**) 0; (**B**) 200; (**C**) 1000; (**D**) 10,000; (**E**) 20,000; and (**F**) 35,000. Insets are ImageJ photos for quantifying the surface porosity. Magnification, 100k×; Scale bar, 500 nm.

**Figure 3:**
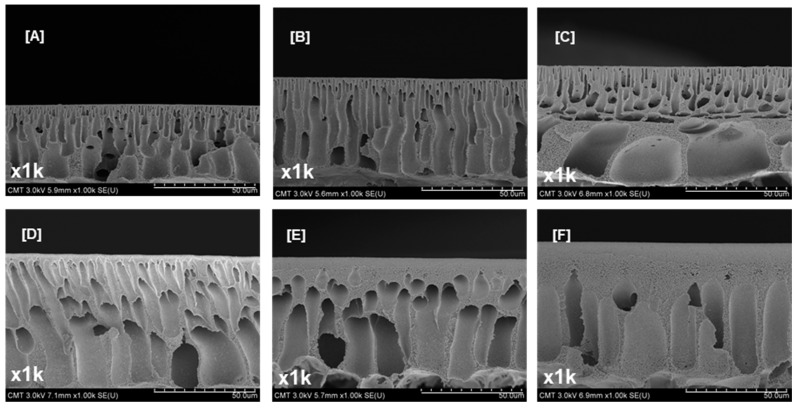
Cross-sectional images of PSf supports modified using PEG of varying molecular weights: (**A**) 0; (**B**) 200; (**C**) 1000; (**D**) 10,000; (**E**) 20,000; and (**F**) 35,000. Magnification, 1k×; Scale bar, 50 µm.

The presence of PEG in PSf/NMP solutions affects the thermodynamics and kinetics of phase-inversion processes (water as a coagulation bath). PEG in dope solutions consumes some solvent, making the polymer concentration and viscosity high―the casting solution becomes thermodynamically less stable, resulting in rapid instantaneous demixing when the cast film is immersed in a coagulation bath. The hydrophilic nature of PEG influenced the rate of exchange between solvent and nonsolvent, as well as the precipitation kinetics and morphology during phase-inversion processes [59]. Figure 2 and Figure 3 demonstrate the effect of varying PEG *M*_W_ on the PSf morphology. The PSf supports were asymmetric—a dense skin layer sat on top of a porous sublayer containing macrovoids.

As shown in Figure 2, increasing the PEG *M*_W_ from 0 to 35,000 g/mol reduced the number of pores on the membrane surface. The surface porosity and pore size are listed in Table 2. Varying the PEG *M*_W_ from 0 to 35,000 g/mol resulted in decreasing the surface porosity and surface pore size from 17.3% ± 0.7% to 8.6% ± 1.0% and from 16.6 ± 2.7 to 11.9 ± 2.6 nm, respectively. Table 1 indicates that viscosity increased with molecular weight. Therefore, during the phase-inversion process, the polymer residence time on the surface was longer. The hydrophilic nature of PEG caused the surface to be dense because of the rapid instantaneous demixing and precipitation of the polymer matrix [59].

Figure 3 displays the cross-sectional SEM images of PSf supports containing different PEGs whose *M*_W_ were varied. Macrovoids in the cross-section were suppressed, and the skin layer was thick because of the high casting solution viscosity (which was associated with high PEG *M*_W_, as indicated in Table 1). The total membrane and skin layer thicknesses (Table S1, see Supplementary Materials) increased from 41.6 ± 4.6 to 66.8 ± 4.1 µm and 452.8 ± 20.6 to 18,318.6 ± 1030.2 nm, respectively. PEGs with a high *M*_W_ slowed down the precipitation kinetics during the phase inversion because of a high-viscosity polymer solution—resulting in a dense surface and thick total membrane and skin layers [59]. The PEG *M*_W_ did not affect the bulk porosity (Table S1, see Supplementary Materials, 82.0–86.2% range). A low-*M*_W_ PEG could easily be removed from the PSf support. However, a high-*M*_W_ PEG tended to entangle with the PSf chains; thus, PEG molecules agglomerated during the phase inversion. PEG when removed might leave large pores in the bulk membrane.

The mobility PEG molecules is lower at higher *M*_W_; thus, they tend to be entangled in the PSf support more easily. When the PEG *M*_W_ was varied from 0 to 35,000 g/mol, the water contact angle (Table 3) was reduced from 83.7° ± 0.3° to 51.0° ± 0.5° (hydrophilicity was enhanced) because of the entanglement of PEG during the PSf precipitation. The effect of adding a high-*M*_W_ PEG is similar to that of increasing the PSf concentration, except that the hydrophilicity is poor without PEG. Increasing the PSf concentration and incorporating high-*M*_W_ PEG result in increase in viscosity (Table 1). If the solution viscosity is high, the formed surface is smooth because the demixing is delayed. Adding PEG of different *M*_W_ resulted in a smooth membrane surface (Table 3) because of the decreased demixing rate of the polymer solution [51]. Water contact angle is affected by surface roughness and the presence of surface functional groups. Generally, increasing the surface roughness enhances hydrophilicity because of the corresponding increase in the surface area. However, our data on surface roughness vs. hydrophilicity showed the opposite result (i.e., increasing the surface roughness adversely affected the hydrophilicity). Therefore, it is reasonable to conclude that the hydrophilic property of PSf supports containing high-*M*_W_ PEG is associated with the PEG hydroxyl functional groups. The presence of several OH groups from PEG is the major factor that causes the change in hydrophilicity (indicated by a high change in contact angle).

**Figure 4:**
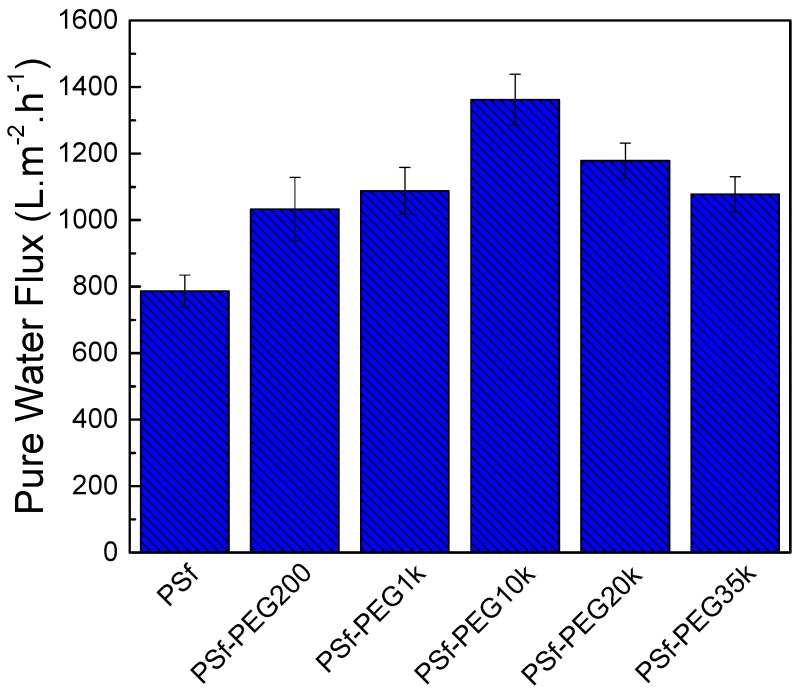
Water flux of PSf supports prepared using PEG of varying molecular weights at 0.6 MPa.

A cross-flow filtration at 0.10 MPa and at 0.6 was performed at room temperature. The flow rate was controlled between 0.60 and 0.70 L·min^−1^. As indicated in Figure 4, the optimum water permeability (1361.9 L·m^−2^·h^−1^·bar^−1^) was attained at PEG10k. A *M*_W_ higher than 10,000 g/mol resulted in lower water permeability because of the thicker skin layer (Figure 3). Despite the increase in the membrane thickness, the PEG-modified PSf still exhibited higher water permeability than the pristine PSf because of the enhanced hydrophilicity.

#### 3.2.2. TFC Membrane Properties

**Figure 5:**
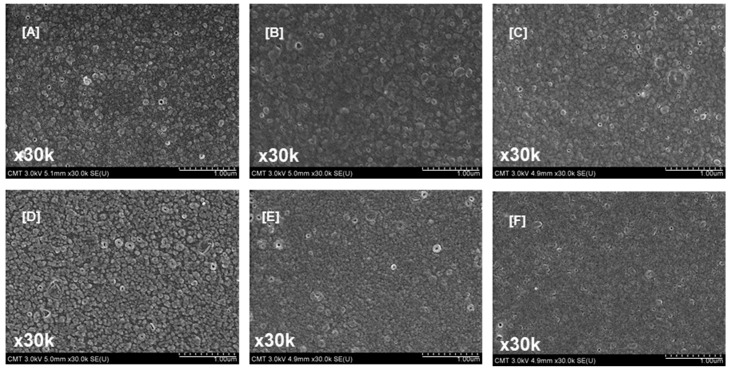
Surface images of thin-film composite membranes prepared using PEG of varying molecular weights: (**A**) TFC–0; (**B**) TFC–PEG200; (**C**) TFC–PEG1k; (**D**) TFC–PEG10k; (**E**) TFC–PEG20k; and (**F**) TFC–PEG35k. Magnification, 30k×; Scale bar, 1 µm.

PIP-based polyamide membranes comprised dense and discrete nodular structures [61]. The average nodule size was estimated to be around 50–100 nm. However, protuberances were different when IP was performed on different PSf membranes (Figure 5). The SEM images illustrate that the nodular structure was larger when the PEG *M*_W_ was lower than 10,000 g/mol. Kwak et al. [62] reported that larger nodular structures represented higher degrees of cross-linking between aqueous and organic monomers. This high cross-linking is due to the presence of more diamines in the PSf support. Increasing the PEG *M*_W_ resulted in small nodules and a smooth membrane surface. These results are related to the membrane support surface pore size and porosity. The membrane prepared using PEG with a higher *M*_W_ provided a smaller surface pore size and lower porosity; thus, the formed polyamide was uniform.

**Figure 6:**
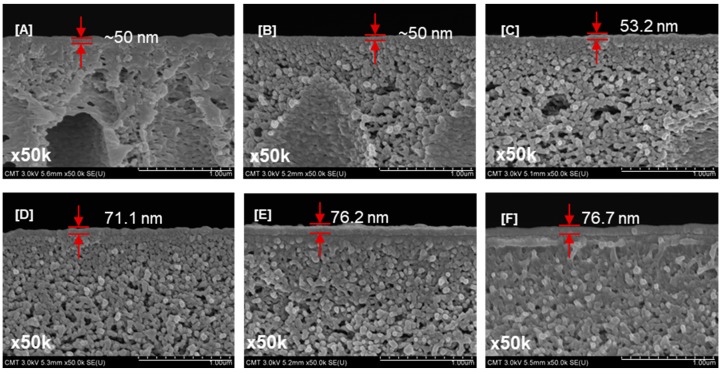
Cross-sectional images of thin-film composite membranes prepared using PEG of varying molecular weights: (**A**) TFC–0; (**B**) TFC–PEG200; (**C**) TFC–PEG1k; (**D**) TFC–PEG10k; (**E**) TFC–PEG20k; and (**F**) TFC–PEG35k. Magnification, 50k×; Scale bar, 1 µm.

Figure 6 shows the cross-sectional images of TFC membranes prepared using different PEG *M*_W_. Increasing the PEG *M*_W_ (0–35,000 g/mol) led to thicker polyamide layers (70.0–76.7 nm). These results correspond with the membrane support surface pore size. As reported in a previous study [49], small pores in the membrane support surface may hinder the penetration of polyamide in the pores. Therefore, the polyamide layer was formed mainly on the membrane surface.

The water contact angles (Table 4) were 36.1°, 38.3°, 29.6°, 25.5°, 21.5° and 16.9° when the PEG *M*_W_ were 0, 200, 1000, 10,000, 20,000 and 35,000 g/mol, respectively. These results indicate that when a high-*M*_W_ PEG was used, the formed polyamide had more carboxyl groups (from ABA and the hydrolysis of TMC) on the membrane surface (i.e., water contact angles were lower). These carboxyl groups were responsible for the increase in hydrophilicity. The surface hydrophilicity of the TFC membrane was not affected by the surface roughness. When the PEG *M*_W_ increased from 200 g/mol to 35,000 g/mol, the surface roughness decreased from 11.6 ± 1.0 nm to 6.9 ± 0.5 nm (Table 4). These results agree with the SEM surface data (Figure 5). There is no correlation between surface roughness and water contact angle. Therefore, the polyamide layer hydrophilicity was dominated by the surface functional groups.

**Figure 7:**
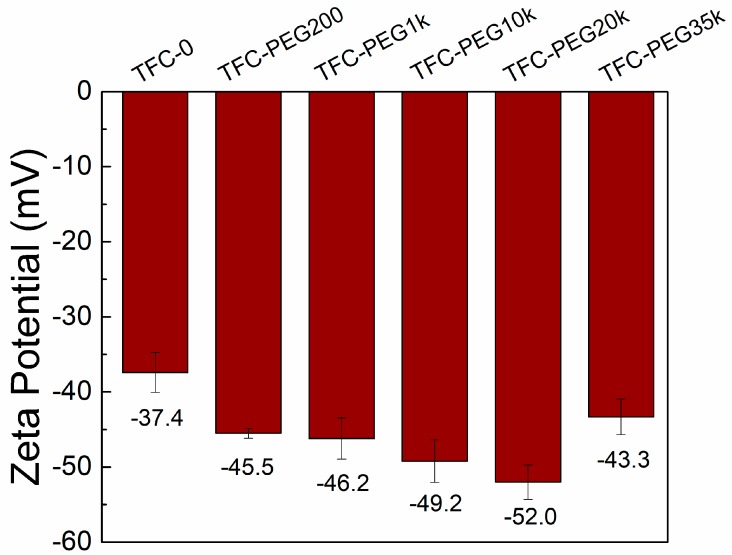
Zeta potential of thin-film composite membranes prepared using PEG of varying molecular weights; pH = 7.0.

Figure 7 compares the zeta potential of TFC membranes prepared using PEG of different *M*_W_ at pH = 7. As expected, the TFC membranes had negatively charged surfaces. The zeta potential decreased from −37.4 to −52.0 mV when the PEG *M*_W_ was increased from 0 to 20,000 g/mol, indicating the presence of more hydrophilic carboxyl groups from the hydrolysis of TMC and from the ABA additive. When the PEG *M*_W_ was 35,000 g/mol, the zeta potential increased to −43.3 mV. Increasing the *M*_W_ of PEG from 0 to 20,000 g/mol resulted in a less porous membrane support. As such, the adsorption of amines was less, leading to a weak cross-linking in the polyamide layer. However, increasing the PEG *M*_W_ to 35,000 g/mol did not affect the membrane support porosity but decreased the water contact angle; thus, the support adsorbed more amines and induced a highly cross-linked polyamide layer.

#### 3.2.3. Separation Performance Test

**Figure 8:**
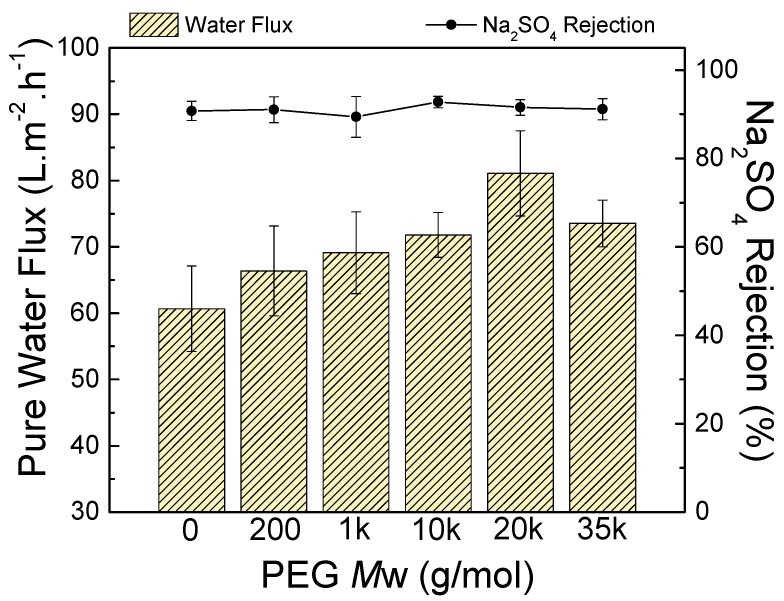
Nanofiltration performance of thin-film composite membranes prepared using PEG of varying molecular weights. Feed = 1000 ppm aqueous Na_2_SO_4_ solution. Operating conditions = 0.60 MPa and 25 °C.

The TFC membrane separation performance was measured at 0.60 MPa; the feed was 1000 ppm aqueous Na_2_SO_4_ solution. Figure 8 compares the NF performance of TFC membranes prepared using PEG of varying *M*_W_. The results reveal that the salt rejection was approximately 91–93%. This indicates that the salt rejection is not affected by modifying the support layer property. These results are similar to the work by Misdan et al. [51], where the salt rejection was not affected when the PSf doped-solution concentration was changed. Without the addition of PEG, the water flux was 60.7 L·m^−2^·h^−1^. Increasing the PEG *M*_W_ from 200 to 20,000 g/mol increased the flux from 66.4 to 81.1 L·m^−2^·h^−1^. This increase is due to the low membrane support surface porosity, diminished surface pore size, and appropriate hydrophilicity; these properties are all associated with high-MW PEG in the PSf support. This PSf support characteristic prevented the polyamide from being trapped in the PSf pores, leading to the formation of a defect-free polyamide layer; consequently, the flux obtained was high. In addition, the water contact angle and zeta potential of TFC membranes decreased; therefore, the water flux increased.

The flux of TFC–PEG35k (support contact angle = 51° ± 0.1°) was lower than that of TFC–PEG20k (support contact angle = 55.5° ± 0.3°). Because of the lower water contact angle of the support, the adsorption of diamine monomer was greater, resulting in a thicker polyamide layer (Figure 6F). Therefore, TFC–PEG20k delivered the highest water flux. A suitable hydrophilicity, low surface porosity, and small pore size of the PSf support favored the formation of a defect-free polyamide layer. The deposition of polyamide in the pores of PSf was prevented, leading to high water flux.

**Figure 9:**
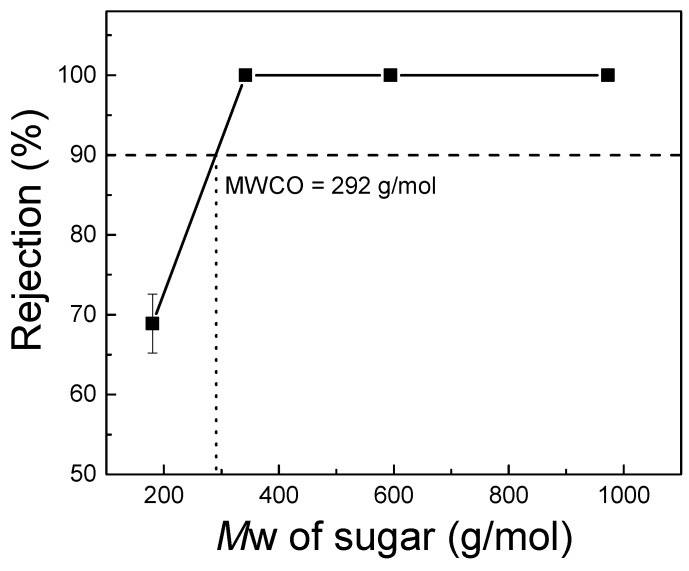
Determination of the molecular weight cutoff of TFC–PEG20k. Feed = 1000 ppm aqueous sugar solution. Operating conditions = 0.60 MPa and 25 °C.

Figure 9 depicts the determination of the TFC–PEG20k molecular weight cutoff. The rejection of sucrose, raffinose, and α-cyclodextrin was 100%, whereas that of glucose was only 69% ± 11.0%. At 90% rejection, the molecular weight cutoff was 292 g/mol. Therefore, this result shows that the membrane molecular weight cutoff was validated to be in the range for nanofiltration.

**Figure 10:**
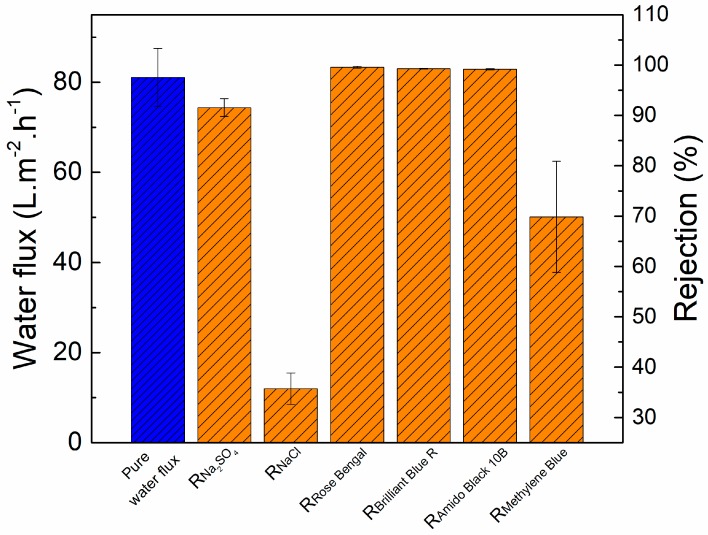
Separation performance of TFC-PEG20k. Feed = 1000 ppm aqueous salt solution or 50 ppm aqueous dye solution. Operating conditions = 0.60 MPa and 25 °C.

Figure 10 illustrates the separation performance of TFC–PEG20k, which was tested on salts or dyes. The rejection of NaCl was only 35.7% ± 3.1%, indicating that this membrane is a nanofiltration membrane, because the rejection of the monovalent ion was lower than 50%. Rose bengal (*M*_W_ = 973.87 g/mol), brilliant blue (*M*_W_ = 825.97 g/mol), and amido black 10B (*M*_W_ = 616.49 g/mol) were all negatively charged dyes and their molecular weights were greater than the molecular weight cutoff of 292 g/mol. Therefore, the TFC–PEG20k had a 99% rejection of negatively charged dyes. However, the rejection of methylene blue (positively charged dye, *M*_W_ = 319.85 g/mol) was only 69.9% ± 11.0%. This was because the TFC–PEG20k had a negatively charged surface. Positively charged substances are adsorbed on the membrane surface; some of them tend to pass through the membrane and are collected in the permeate.

### 3.3. Mechanism

**Figure 11:**
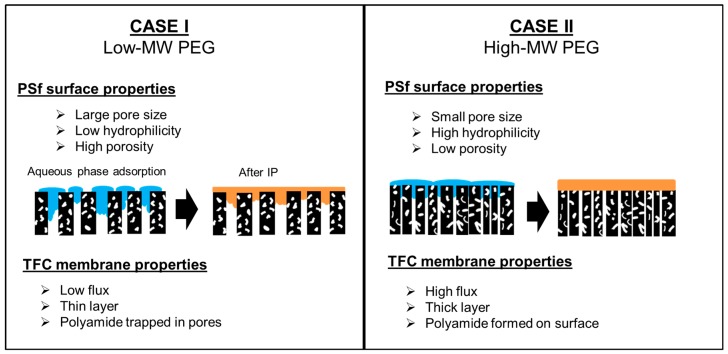
Schematic representation of polyamide formation on two kinds of PSf supports.

The type of PEG-doped PSf supports would depend on the PEG *M*_W_. Figure 11 demonstrates two cases of polyamide formation on different types of PSf support. In Case I, the support was highly porous but had low hydrophilicity. Although the hydrophilicity was low, water penetrated because of the large pores. Consequently, during IP, some polyamide formed in the pores despite the extreme thinness of the polyamide layer. This condition caused high water resistance. For Case II, the PSf support had high hydrophilicity, and its pore size and surface porosity were small. During IP, the formation of a defect-free polyamide layer was mainly on the surface. In other words, no polyamide formed in the pores. Therefore, the flux in Case II was higher than that in Case I because the polyamide did not block the pores.

## 4. Conclusions

TFC NF polyamide membranes were fabricated using various PEG-modified PSf supports. PEG additives altered the surface functional groups, surface porosity, surface pore size, total membrane and skin layer thicknesses, water contact angle, and water permeability of PSf supports. Generally, increasing the PEG *M*_W_ decreased the membrane surface porosity, pore size, and water contact angle. These results are due to the entrapment of PEG in the PSf support. PSf supports exhibiting high water permeability was not a prerequisite for the corresponding fabricated TFC membranes to have high flux as well. Having a low surface porosity and a small surface pore size, the membrane support prevented the formation of polyamide layer in the PSf pores. A high hydrophilicity guaranteed adsorption of enough monomers despite small pores on the membrane surface. PSf supports exhibiting a low surface porosity, small surface pore size and suitable hydrophilicity favored the formation of a polyamide layer with high water flux. The TFC polyamide membrane fabricated using PSf-PEG20k delivered the best NF performance (flux = 81.1 ± 6.4 L·m^−2^·h^−1^ and R_Na2SO4_ rejection = 91.5% ± 1.8%, R_NaCl_ = 35.7% ± 3.1%, MWCO = 292 g/mol). The membrane support with a low surface porosity (9.5% ± 1.1%), small surface pore size (10.3 ± 3.0 nm), suitable hydrophilicity (55.5° ± 0.3°), and low surface roughness (4.2 ± 0.2 nm) is recommended to be suitable for fabricating high-performance TFC NF membranes. The investigation of the effects of substrate properties on the membrane antifouling ability would be our future work.

## Figures and Tables

**Table 1:** Composition and viscosity of casting solutions.

Membrane	Solution Composition (wt %)			PEG *M*_W_ (g/mol)	Viscosity (mPa·s)		
	PSf	NMP	PEG ^a^				
PSf	16	84	-	-	555	±	9
PSf-PEG200	16	84	50	200	552	±	7
PSf-PEG1k	16	84	50	1000	592	±	8
PSf-PEG10k	16	84	50	10,000	850	±	13
PSf-PEG20k	16	84	50	20,000	1106	±	15
PSf-PEG35k	16	84	50	35,000	1546	±	21

^a^ Based on the total amount of PSf.

**Table 2:** Physical characteristics of PSf supports prepared using PEG of varying molecular weights.

Membrane	Surface Porosity ^a^ (%)			Surface Pore Size ^a^ (nm)		
PSf	17.3	±	0.7	16.6	±	2.7
PSf–PEG200	14.5	±	0.9	12.2	±	2.3
PSf–PEG1k	11.2	±	0.8	10.8	±	3.4
PSf–PEG10k	10.9	±	1.0	10.4	±	2.7
PSf–PEG20k	9.5	±	1.1	10.3	±	3.0
PSf–PEG35k	8.6	±	1.0	11.9	±	2.4

^a^ Analyzed using ImageJ software.

**Table 3:** Water contact angle and surface roughness of PSf supports prepared using PEG of varying molecular weights.

Membrane	Water Contact Angle (°)			Surface Roughness, *R*q (nm)		
PSf	83.7	±	0.3	6.6	±	0.6
PSf–PEG200	73.1	±	0.4	6.6	±	0.2
PSf–PEG1k	69.1	±	0.6	6.5	±	0.3
PSf–PEG10k	60.0	±	0.5	4.6	±	0.1
PSf–PEG20k	55.5	±	0.3	4.2	±	0.2
PSf–PEG35k	51.0	±	0.5	4.1	±	0.2

**Table 4:** Water contact angles and surface roughness of TFC membranes prepared using PEG of varying molecular weights.

Membrane	Water Contact Angle (°)			Surface Roughness, *R*q (nm)		
TFC–0	36.1	±	1.2	8.0	±	0.1
TFC–PEG200	38.3	±	0.7	11.6	±	1.0
TFC–PEG1k	29.6	±	0.9	10.2	±	1.4
TFC–PEG10k	25.5	±	0.8	7.8	±	0.6
TFC–PEG20k	21.5	±	0.6	6.9	±	0.8
TFC–PEG35k	16.9	±	1.0	6.9	±	0.5

## Supplementary Materials

The following is available online at www.mdpi.com/2073-4360/9/10/505/s1, Table S1: Physical characteristics of PSf support prepared using PEG with varying molecular weights.
