# Supplementary material for: Correlating PSf Support Physicochemical Properties with the Formation of Piperazine-Based Polyamide and Evaluating the Resultant Nanofiltration Membrane Performance
Source: Polymers (Basel). 2017 Oct 13;9(10):505. doi: 10.3390/polym9100505 (PMC6418895; doi:10.3390/polym9100505)
Supplement: Supplementary file 1 [file polymers-09-00505-s001.pdf]

Polymers

Electronic Supporting Information

Correlating PSf Support Physicochemical Properties with the Formation of Piperazine-Based Polyamide and Evaluating the Resultant Nanofiltration Membrane Performance

Micah Belle Marie Yap Ang[1], Victor Jr. Lau[2], Yan-Li Ji[1,3*] Shu-Hsien Huang[1,4*], Quan-Fu An[5], Alvin R. Caparanga[2], Hui-An Tsai[1], Wei-Song Hung[1], Chien-Chieh Hu[1], Kueir-Rarn Lee[1*], and Juin-Yih Lai[1,6]

[1] R&D Center for Membrane Technology, Department of Chemical Engineering, Chung Yuan University, Taoyuan 32023, Taiwan; mbmyang@gmail.com (M.B.M.Y.A.); huian@cycu.edu.tw (H.A.T.); wshung@cycu.edu.tw (C.C.H.); jylai@cycu.edu.tw (J.Y.L.)

[2] School of Chemical Engineering and Chemistry, Mapúa University, Manila 1002, Philippines; victorlau@yahoo.com (V.J.L.); arcaparanga@mapua.edu.ph (A.R.C.)

[3] Center for Membrane and Water Science & Technology, Ocean College, Zhejiang University of Technology, Hangzhou 310014, China;

[4] Department of Chemical and Materials Engineering, National Ilan University, I-Lan 26047, Taiwan 2

[5] Beijing Key Laboratory for Green Catalysis and Separation, College of Environmental and Energy Engineering, Beijing University of Technology, Beijing 100124, China; anqf@bjut.edu.cn (Q.F.A.)

[6] Department of Chemical Engineering, National Taiwan University of Science and Technology, Taipei 10607, Taiwan

* Correspondence: krlee@cycu.edu.tw; Tel.: +886-3-2654190

huangsh@niu.edu.tw; Tel.: +886-3-9317505

yanliji@zjut.edu.cn; Tel.: +86-571-88324135

The bulk porosity lists in the Table 1S. The PEG MW did not affect the bulk porosity (82.0–86.2 % range). For low-MW PEG, the majority of PEG was readily removed during the phase-inversion process, leaving behind a small amount of PEG. However, for high-MW PEG, the majority of PEG was retained in the support, and although the amount of PEG removed was smaller (compared with the case of low-MW PEG), its MW was higher, leading to the formation of bigger pores. Therefore, the effect of PEG MW on the bulk porosity was not substantial.

Table S1. Physical characteristics of PSf support prepared using PEG with varying molecular weights.

Membrane	Bulk porosity (%)			Total membrane thickness[a] (μm)			Skin layer thickness[b] (nm)		
PSf	85.8	±	1.9	41.6	±	4.6	452.8	±	20.6
PSf-PEG200	86.2	±	2.3	48.1	±	2.8	719.2	±	16.1
PSf-PEG1k	82.0	±	2.1	51.7	±	5.7	860.9	±	33.6
PSf-PEG10k	85.2	±	2.0	62.9	±	2.4	2569.9	±	204.1
PSf-PEG20k	86.8	±	0.7	63.8	±	6.7	7466.8	±	300.3
PSf-PEG35k	85.5	±	2.0	66.8	±	4.1	18318.6	±	1030.2

[a]excludes nonwoven layer.
[b]measured from surface to macrovoid tip.
